# Telework satisfaction, wellbeing and performance in the digital era. Lessons learned during COVID-19 lockdown in Spain

**DOI:** 10.1007/s12144-022-02873-x

**Published:** 2022-02-20

**Authors:** Joanna Blahopoulou, Silvia Ortiz-Bonnin, Maribel Montañez-Juan, Gema Torrens Espinosa, M. Esther García-Buades

**Affiliations:** Department of Psychology, University of the Balearic Islands (UIB), Ed. Guillem Cifre de Colonya, A-210. Ctra.Valldemossa, km 7.5. 07122, Palma de Mallorca, Spain

**Keywords:** Telework, Working from home (WFH), Satisfaction, Wellbeing, Performance, COVID-19, Children

## Abstract

This study used a prospective design to examine the effects of telework satisfaction (time 1) on subjective wellbeing and self-reported performance (time 2) during the COVID-19 lockdown. Data were collected from 111 teleworkers through an online survey the first weeks of strict lockdown in Spain. Telework satisfaction showed positive direct effects on both subjective wellbeing and self-reported performance. Further, subjective wellbeing partially mediated the relationship between telework satisfaction and self-reported performance. Interestingly, employees with children felt less telework satisfaction but higher subjective wellbeing. The novelty of this study is that we evaluate the level of satisfaction with telework using a specific set of items that assess the employees’ contentment with diverse telework facets. Given the spreading of telework and the increasing competitiveness of organizations, we discuss practical implications in times of crisis, both present and future.

## Introduction

At the beginning of 2020 it was hard to imagine that a drastic change in our lives was about to come through a worldwide public health crisis due to a virus (COVID-19). In the absence of knowledge on adequate treatment or a vaccine, most countries enforced self-isolation measures to stop the spread of the disease and release over-burdened health systems. The Spanish Government declared the state of alert and a 14 days’ nationwide lockdown on March 16^th^, which was extended four times and ended on the 20^th^ of June 2020 (RDL 463/2020, March 14^th^). Telework was adopted in organizations as a temporary working measure to ensure the continuity of private businesses and public administration services (Amankwah-Amoah et al., 2020; Anker, 2021; Belzunegui-Eraso & Erro-Garcés, 2020; Bhumika, 2020; Palumbo, 2020; Tavares et al., 2020). Thousands of employees and managers started to work from home (WFH) in this uncertain situation (Kirchner et al., 2021). Employees teleworking during lockdown were facing two big challenges. The first challenge was to handle emotional suffering because of infection fear, job insecurity, dismissals menace, financial problems and the multiple effects of the crisis that were threatening their wellbeing. The second challenge was to rapidly adjust to a new work environment and work demands in a matter of days (in Spain, from a Friday at work to Monday with telework) without any preparation, planning or training time, sourcing from the IT equipment available at home, struggling between work and family demands, and trying to maintain high work performance.

Research about the effects of telework (before COVID-19) on employees’ wellbeing is inconclusive (Joyce et al., 2010) and findings about the relationship between telework and performance are contradictory (Giménez-Nadal et al., 2019; Solís, 2017). Most studies are cross-sectional with one time point of data collection and compare teleworkers with office workers without analyzing differences in working characteristics and conditions (Vander Elst et al., 2017). Thus, the discrepancy of the findings leads to the assumption that there is a need for in-depth, longitudinal studies that analyze the conditions under which the positive or negative effects of telework take place (Solís, 2017). Furthermore, scholars in this field propose to study distinctive telework circumstances and search for moderating and mediating variables (Karanikas & Cauchi, 2020; Solís, 2017; Vander Elst et al., 2020).

Before COVID-19, there was no study investigating telework adoption and its effects in an epidemic context (with no damage to physical or technological infrastructures) and only a few studies in a crisis specific context (Carillo, 2020). Examples are the study after September 11^th^ 2001 (Mello et al., 2011) and the research following the Christchurch earthquakes in 2010–2011, which found that moving to WFH following a natural disaster supported business continuity and employee wellbeing (Donnelly & Proctor-Thomson, 2015; Green et al., 2017).

Research on telework during COVID-19 lockdown has focused on facilitating factors (Belzunegui-Eraso & Erro-Garcés, 2020), description of telework (number of hours, easiness, difficulties; Tavares et al., 2020; Morilla-Luchena, et al., 2021), advantages and disadvantages (Ipsen et al., 2021), and its effects on work-life balance (Bhumika, 2020; Palumbo, 2020) or engagement (Miglioretti et al., 2021; Wang & Parker, 2021). However, there is a research gap on the effects of telework satisfaction during COVID-19 on outcomes such as wellbeing and performance. Gaining a better knowledge of telework satisfaction will be more important than ever since it seems that telework came to stay in our lives also after the pandemic (Belzunegui-Eraso & Erro-Garcés, 2020; Wang & Parker, 2021). Thus, research on how employees evaluate telework facets is essential for the present and the future of management and planning of human resources.

Our research question is thus: How did satisfaction with telework affect employee wellbeing and performance in an adverse situation such as the COVID-19 lockdown? This study is innovative in several ways. First, we evaluate the level of satisfaction with telework by assessing the employees’ satisfaction with specific telework features. Doing so we overcome the limitation of studies that compare the satisfaction levels between teleworkers with office workers using general measures of job satisfaction. In this vein, it will help to draw a richer picture of the nature of the satisfaction with telework. Second, we offer a prospective design with two waves of data collection, which allows establishing predictive relationships between employee satisfaction with telework conditions (time 1) and employee outcomes such as subjective wellbeing and self-reported performance (time 2). This is an important contribution because most telework research is cross-sectional (e.g., Wang & Parker, 2021). Third, we shed light about the importance of a positive evaluation of telework and its impact on employee wellbeing and performance in a unique context: the COVID-19 lockdown.

Conducting the study in times of COVID-19 lockdown is valuable for many reasons. It gives us the opportunity to explore the importance of telework satisfaction under adverse conditions. The study of (tele)work satisfaction might impact wellbeing and performance in crisis and non-crisis times. However, we suggest that a positive evaluation of the virtual working conditions might have gained an extraordinary salience for employee outcomes in crisis-times because wellbeing was especially threatened due to social isolating lockdown measures and pandemic related fears (e.g., Agha, 2021). Unfortunately, future pandemics, natural disasters and other crises entailing lockdowns will come, and learning from this extraordinary situation can help us in the future.

## Literature Review

### Conventional vs. Crisis-induced Telework

Telework, also known as working from home (WFH), telecommuting, remote working, e-work or home-based working is a work arrangement, that has become a popular practice due to the advancement in information and communication technologies (ICTs) (Lebopo et al., 2020). While consensus on a unified definition is missing across academic fields, most scholars concur on the main characteristics of this work arrangement: a) employees perform their job tasks while being away from the normal workplace, b) employees use high-technology equipment to work (Baruch, 2000; Carillo et al., 2020; Gajendran & Harrison, 2007). The current paper will use telework and working from home (WFH) indistinctly.

Before the pandemic only 15% of employees were teleworking in Europe, whereas in Spain just about 4% of employees worked regularly from home before the coronavirus crisis (Instituto Nacional de Estadística, 2020). The rate of implementation of flexible working arrangements in Spain was thus one of the lowest in Europe. Besides, many employees were only occasional teleworkers. According to Eurofound (2020) a total of 36.3% of people working in the EU27 started teleworking fulltime as a result of the pandemic.

Till 2019 the factors that influenced the use of telework were divided into individual factors (e.g., work style), job factors (e.g., autonomy, feedback), organizational factors (e.g., management trust, technical support) and home or family factors (e.g., young children) as proposed by the model of Baruch and Nicholson (1997). A new factor brought about by COVID-19, the environment/safety factor, has led to an unforeseen acceleration of the implementation of teleworking practices around the world (Belzunegui-Eraso & Erro-Garcés, 2020; Mallett et al., 2020; Raišiene et al., 2020).

‘Conventional telework’ is quite different from ‘crisis-induced telework’ (Carillo et al., 2020). First, ‘Conventional telework’ is often offered as an instrument to improve struggling between work and life roles by increasing worker flexibility to combine office work with work outside the workplace. ‘Crisis-induced telework’ is teleworking in response to a crisis; it refers to a sudden and mandatory full-time emergency solution. Literature refers to these two characteristics (mandatory and full-time) as two conditions of telework with a decisive impact on employee wellbeing (Karanikas & Cauchi, 2020).

Telework seems to have more advantages when it is a free choice than when people are forced to it. Furthermore, following the ‘sweet spot hypothesis’, employees who telework occasionally experience the best outcomes (Karanikas & Cauchi, 2020). Instead, working from home full-time leads to a reduction of face-to-face contacts with colleagues and supervisors and the loss of direct, personal, emotional, and social support, which is difficult to obtain through virtual interactions (Karanikas & Cauchi, 2020). Implicit in conventional telework is the assumption of working outside the office as a well-planned work practice, that might include a trial or training period, digitalized information, provision of equipment and essential IT tools (internet and intranet-connection etc.).

‘Crisis-induced telework’ due to COVID-19 took place under quite different conditions, often with no preparation time. This was especially dramatic in Spain because most people had no prior telework experience, and did not have proper virtual working conditions at home (e.g., separate working space, availability of technical resources, accessibility to data or files etc.). Moreover, further specific situations deriving directly from the lockdown context might have affected the telework experience as well. Under ‘ordinary’ circumstances children are at school while the parents are working from home. Instead, during lockdown most working parents had to deal with increased childcare demands (e.g., homeschooling, cooking meals, etc.) (Shockley et al., 2021). Secondly, lockdown increased social isolation by reducing social face-to face contact to family members living in the same household (if any). Thirdly, the pandemic might have heightened stress and anxiety (Salari et al., 2020), due to worries that employees and/or their families might suffer from COVID-19 infection, and the anticipation of financial and social consequences of the pandemic (Ebrahimi et al., 2021). These specific circumstances during lockdown could have been at the expense of the employees’ wellbeing and job performance (Ipsen et al., 2021).

Table 1 summarizes the characteristics of ‘conventional telework’ comparing them to the characteristics of ‘crisis-induced telework’ during the first lockdown due to COVID-19.

**Table 1:** Telework characteristics of Conventional vs. Crisis-induced telework during first COVID-19 lockdown

Telework characteristics	
Conventional telework	Crisis-induced telework
• Voluntary	• Mandatory
• All or part of the working hours	• Full-time
• Preparation and Training (digital content and cybersecurity)	• No Preparation
• Adaptation of physical work environment at home, technology access and ICT tools	• (Potential) lack of ICT tools (hard/software, access to internet or intranet)
• Workplace flexibility (somewhere outside the office, not only at home)	• At home
• Children at school	• Children at home
• Social relations	• Social isolation

Previous studies identified a large list of advantages of ‘conventional teleworking’ for the organization (e.g., reduced operating costs, improved productivity due to less work interruptions, improved attendance, and improved attraction and retention of workers), for employees (e.g., less travel costs, less travel time, more flexibility, improved work-life balance, higher job satisfaction) and for society (e.g., less energy consumption, less traffic congestions and reduced air pollution) (Allen et al., 2015; Belzunegui-Eraso & Erro-Garcés, 2020; Blahopoulou, 2012; Giménez-Nadal et al., 2019). Additionally, findings draw attention to some important disadvantages of telework such as psychological isolation (Bartel et al., 2012), increased work-family conflict due to an ‘always-on culture’ characterized by expected availability beyond working hours (Arlinghaus & Nachreiner, 2014; Blahopoulou, 2015; Shepherd-Banigan et al., 2016), and missing promotion and career opportunities because of a lower visibility (Davidson & Khalifa, 2000; Maruyama & Tietze, 2012). Moreover, there are some controversial findings. Some studies report an improved work-life balance of teleworkers, and others report increased work-family conflict (Bellmann & Hübler, 2020). Furthermore, there are studies arguing about improved productivity of teleworkers due to less interruptions and higher concentration, and others report less productivity of teleworkers due to a lack of supervisor support (Charalampous et al., 2019).

In sum, the benefits of ‘conventional teleworking’ in terms of wellbeing and performance seem far from straightforward (Allen et al., 2015; Golden & Veiga, 2008). The evidence regarding the advantages of ‘conventional teleworking’ for employees’ wellbeing and performance remain ambiguous. Further, a recent study analyzed advantages and disadvantages of working from home during COVID-19 for knowledge workers in 29 European countries (Ipsen et al., 2021). The main advantages were better work–life balance, improved work efficiency, and perception of greater work control. The main disadvantages highlighted home office constraints, work uncertainties, and inadequate tools. This evidence suggests that some benefits of ‘conventional telework’ (i.e., better concentration, fewer interruptions) may have disappeared, and some disadvantages may have increased (i.e. psychological isolation) during COVID-19 lockdown.

Thus, our purpose is to shed light on the relationship between telework satisfaction and two outcome variables (subjective wellbeing and self-reported performance), that are equally interesting for employees and organizations. Keeping in mind that ‘crisis-induced telework’ was quite different than ‘conventional telework’ raises the questions: Why study telework during lockdown? And what can we learn for the implementation of telework after the pandemic? Exploring the effects of ‘crisis-induced telework’ can help us extract lessons both for potential future crisis-contexts (or future pandemic waves) but also for ‘conventional telework’ with similar conditions (i.e. mandatory and fulltime telework in non-crisis times or telework with children at home). In the following sections we present our theory-based hypotheses.

### Telework Satisfaction and Wellbeing

Past research has associated telework with higher levels of wellbeing (Anderson et al., 2015; Kossek et al., 2006) and lower levels of strain (Bentley et al., 2016). Research in this area has mainly focused on comparing the benefits for teleworkers and office-based workers (Fonner & Roloff, 2010). Recent studies go a step further and start focusing on characteristics of teleworking and the conditions under which it is implemented (for example, number of days working from home a week) (Vander Elst et al., 2017). Following this trend, we focused on telework satisfaction, which refers to the positive evaluation of different telework facets (e.g., social support, IT equipment). Only few previous studies have tried to use specific items to measure telework satisfaction (Baker, 2007; Staples, 1999).

Several meta-analyses confirm a positive relationship between job satisfaction and wellbeing (Bowling et al., 2010; Thoresen et al., 2003). But the nature of the causal relationship is still unclear. Some studies contend that job satisfaction precedes wellbeing (e.g., Chacko, 1983), and for instance Judge and Watanabe (1993) contend it could be either an antecedent or both variables could present a reciprocal relationship. According to the part-whole theory or spillover hypothesis (Bakker & Demerouti, 2013; Sironi, 2019), job satisfaction (in our case telework satisfaction) is one of the determinants of subjective wellbeing because specific life domains are critical in affecting the general wellbeing of individuals. In other words, positive experiences at work have a positive influence in other non-work life spheres, which in turn enhance wellbeing. Extending these arguments to telework satisfaction, we suggest that employees who were satisfied with their teleworking situation during the lockdown period would present higher levels of subjective wellbeing. The novelty of our study is that we measure satisfaction with telework conditions and not general job satisfaction.

Our research question is whether satisfaction with telework is beneficial for employees in a context where the main advantages of teleworking (e.g., more flexibility and less work interruptions) are missing. We suggest that despite the crisis-induced teleworking features (e.g., mandatory and fulltime) telework satisfaction will be beneficial for employee wellbeing. Further, we propose two reasons that explain why telework satisfaction is expected to increase the employees’ levels of wellbeing. First, in times of crisis, working can become a coping strategy to adjust to the new situation (Kirchner et al., 2021) and to promote mental health and resilience (Heir et al., 2021). In their study after the Oslo terrorist attack in 2011, Heir et al. (2021) found that work provided employees with a sense of cohesion, supportive management, and peer support, thus enhancing their wellbeing. In face of COVID-19, the organizations’ facilitation of adequate telework conditions may have helped employees to cope with the new situation. Second, according to social comparison theory (Festinger, 1954; Páez Gabriunas, 2010), teleworkers that are satisfied with their working conditions compare themselves with people in worse conditions (e.g. essential workers who had to go to work being exposed to the virus), and as a result present high levels of wellbeing.

Based on the previous arguments and findings, we propose that telework satisfaction will increase the employees’ levels of wellbeing during lockdown. Thus, we hypothesize:

- H1. Telework satisfaction (T1) will predict subjective wellbeing (T2)

### Telework Satisfaction and Performance

Previous research has shown that telework is frequently claimed to enhance performance (Baker et al., 2007; Golden & Veiga, 2008), and teleworkers commonly report increases in their own perceived productivity (Baruch, 2000). There are several reasons why telework may improve performance and are related to the conditions in which telework is organized. For instance, telework may lead to improved productivity because it allows higher levels of concentration, less interruptions, and higher perceived control over work (Belzunegui-Eraso & Erro-Garcés, 2020; Bosua et al., 2013; Giménez-Nadal et al., 2019; Karanikas et al., 2020; Nakrošienė et al., 2019).

The relationship between job satisfaction (in our case telework satisfaction) and job performance has been called the Holy Grail of organizational research (Wright & Cropanzano, 2007). Different theoretical frameworks have been applied to explain why job satisfaction would increase performance. For instance, the general attitude-behavior link at the base of the Happy-Productive worker thesis suggests that people satisfied with their job would be willing to undertake positive behaviors towards their task, colleagues, and organization, therefore improving productivity (Judge et al., 2001). Social exchange theory suggests that when workers are satisfied with their job and organization, they will be willing to reciprocate to the organization by being productive (Blau, 1964; Ostroff, 1992). Recent meta-analyses have demonstrated a positive correlation between job satisfaction and job performance both in cross-sectional (Harrison et al., 2006) and longitudinal studies (Alessandri et al., 2017; Riketta, 2008).

In summary, previous evidence supports the idea that telework improves perceived performance in a normal situation. Besides, research has supported that employee satisfaction leads to increased performance through positive behaviors which help individual and collective productivity (García-Buades et al., 2020; Warr & Nielsen, 2018). Typical benefits of conventional telework were missing during lockdown. For example, Wang and Parker (2021) showed that working from home during lockdown meant more interruptions by the family, which in turn negatively affected work effectiveness. However, Ipsen et al., (2021) found that people who teleworked during lockdown improved work efficiency. One explanation for these contradictory results may be related not to the very fact of teleworking or not, but instead whether teleworking is satisfactory or not. Overall, given the theoretical and empirical evidence we contend that satisfaction with telework leads to positive performance outcomes. Thus, we hypothesize:

- H2. Telework satisfaction (T1) will predict self-reported performance (T2)

### Subjective Wellbeing and Performance

Subjective wellbeing is defined as people’s evaluations of their happiness and is usually assessed as the experience of positive affect, absence of negative affect, and life satisfaction (Dolan et al., 2011). Research on wellbeing provides empirical evidence that happy people are more productive (Zelenski et al., 2008). Several characteristics of happy people are related with increased performance. Happy people feel less concerned with negative threats, receive more co-worker and supervisory support, feel they have more control over events, are more optimistic about the future and more proactive etc. (Wright & Cropanzano, 2007). These positive feelings are likely to benefit their own performance and positively impact their colleagues’ performance. Alternatively, a person that is unhappy and emotionally drained is unlikely to be productive and this will negatively affect the performance of their coworkers.

The link between wellbeing and performance has also relied on theories such as the broaden-and-build model of positive emotions, which contends that positive emotions ‘share the ability to broaden people's momentary thought-action repertoires and build their enduring personal resources, ranging from physical and intellectual resources to social and psychological resources’ (Fredrickson, 2001, p.219). For instance, joy may enhance creativity, interest creates the urge to explore and take in new information, or pride creates the urge to share achievements or envision new and greater achievements (Fredrickson, 2001). Building on the previous theoretical arguments, we hypothesize:

- H3. Subjective wellbeing (T2) is positively associated with self-reported performance (T2)

Based on the aforementioned arguments, we propose that telework satisfaction increases subjective wellbeing (Hypothesis 1) and self-reported performance (Hypothesis 2). Further, we propose that increased subjective wellbeing is associated with increased self-reported performance (Hypothesis 3). In addition, we test the mediating role of subjective wellbeing in the telework satisfaction and performance relationship. We propose that satisfaction with telework will have a positive impact on self-reported performance through subjective wellbeing. In other words, employees that positively evaluate telework conditions will present higher levels of wellbeing that in turn will increase their performance. Given our previous arguments suggesting that satisfaction with telework directly affects employee performance, only partial mediation is expected.

Thus, we hypothesize:

- H4. Subjective wellbeing (T2) will partially mediate the relationship between telework satisfaction (T1) and self-reported performance (T2).

## Method

### Study Context

The context in which the study took place was unique for several reasons. The data were gathered during the weeks when Spain was under Europe’s strictest lockdown measures due to very high infection rates (e.g., only essential workers could leave their homes; children were under strict lockdown for 6 weeks). At the time of the survey, little was known about the coronavirus, and fears of infection and doubts about effective treatment were high. This situation was challenging people’s physical and mental wellbeing. Further, the Spanish workforce had low previous telework experience, with only 4% teleworking regularly before COVID-19 (Instituto Nacional de Estadística, 2020). Adapting to sudden unplanned telework and keeping up performance in a stressful situation of a pandemic while confined with the family at home posed an important challenge for telework performance and wellbeing.

### Sample and Procedure

The study sample consisted of 111 Spanish employees working from home during the first COVID-19 lockdown, aged between 25 and 65 years old (Mean = 42.45; SD = 8.34). The majority of respondents were women (80.2%), had a university degree (80.2%), and had children under 18 years old (63.1%). Most employees had an organizational tenure higher than 5 years (68%) and worked from home for the first time (75.7%). The sample was heterogeneous and many sectors were represented: education (25.2%), administration (15.3%), health (9%), tourism and hospitality (7.2%), IT, commerce, and construction (4.5% for each sector) and industry, transportation, and security (3.6% each). Employees from the public sector (23.4%), the private sector (64.4%), and others (11.7%) participated in the study.

The objective was to explore the early experiences with working from home during COVID-19. We created a questionnaire using the free online platform Google forms. We used a non-probabilistic snowball sampling method to recruit participants across different occupations and locations in Spain, similar to many other studies during COVID-19 lockdown (e.g., Chong et al., 2020; Lin et al., 2021). We shared the link through social media networks (e.g., LinkedIn), published it on the website of the researchers’ University, and through personal and professional contacts who were asked to distribute it further.

A time-lagged design was chosen for the study to allow for a richer picture on telework outcomes and fill the gap on time-lagged and longitudinal studies about telework (Charalampous et al., 2019). Two waves of data were collected with a time lag of one month between time 1 (April 1^st^, 2020) and time 2 (May 1^st^, 2020). The time lag was short because the lockdown was planned initially for two weeks only, although after a few extensions it finally lasted three months. Among the respondents of the T1 online survey (N = 456 people), 118 people met the inclusion criteria (i.e., teleworking) and voluntarily agreed to participate in the T2 survey. The final sample consisted of 111 participants who completed both surveys and were included in our analysis. Following ethical guidelines, all participants received a short video with descriptive results and a thank you message after finishing the data collection period.

### Measures

The questionnaire included measures of telework satisfaction (T1), subjective wellbeing and self-reported performance (T2), and socio-demographic variables.

#### Telework Satisfaction

Telework satisfaction refers to an affective evaluative response towards teleworking. Previous research on telework has mostly relied on measures of global job satisfaction to compare the results of occasional teleworkers with non-teleworkers (Charalampous et al., 2019). Only few researchers have used scales tapping into satisfaction with the unique working conditions of teleworkers (Baker et al., 2007; López Araújo & Osca Segovia, 2008; Staples et al., 1999). For instance, Staples et al. (1999) assessed satisfaction with facets such as physical work conditions, management, work hours, and job variety.

Scales with facets allow for a better understanding and diagnose on how to improve satisfaction with telework. Thus, building on Staples et al.'s work (1999), we measured telework satisfaction through a nine-item scale (three items adapted from Staples, and six new items based on an in-depth literature review). The facets included were amount of work, type of work, organization of working hours, possibility to concentrate without interruptions, family/partner respect of time and workspace, supervisor support, colleague support, availability of information communication technology (ICT), and physical conditions. Sample items were ‘During the past week I am satisfied… with the ICT tools I have to work from home’, ‘…with the possibility to concentrate on work without interruptions.’ The response options were a six-point scale ranging from 1 (very unsatisfied) to 6 (very satisfied). Cronbach’s alpha was 0.84.

#### Subjective Wellbeing

We measured the degree of subjective wellbeing (affective wellbeing) over the past two weeks through the WHO-5 (World Health Organization—Five Wellbeing Index) in its Spanish version (Topp et al., 2015). This Index is a short and generic global self-reported measure of current mental wellbeing. It consists of 5 items; a sample item is ‘Over the past 2 weeks… I have felt cheerful and in good spirit’. The response options were a six-point scale ranging from 0 (at no time) to 5 (all of the time). Cronbach’s alpha reliability coefficient was 0.84. To obtain a WHO-five wellbeing score, the answers to the five items are added (raw score), and the sum is multiplied by four (percentage score, ranging from 0 to 100). A percentage score of 0 represents the worst imaginable wellbeing, whereas a score of 100 represents the best imaginable wellbeing. Following the WHO-5 guidelines, a percentage score below 52 (a raw score below 13) indicates poor wellbeing and is an indication for testing for depression under ICD-10 (Major Depression Inventory).

#### Self-reported Performance

We asked participants to rate their task performance in the past two weeks. The scale consisted of three items to rate the quantity of work, the quality of work, and the achievement of work goals (based on González-Romá & Gamero, 2012; Koopmans et al., 2012). A sample item is ‘How do you rate the quality of your own work in the past two weeks?’. The response options were a five-point scale ranging from 1 (very bad) to 5 (very good). Cronbach’s alpha for self-reported performance was 0.85.

### Control Variables

Based on previous research, we included control variables which may affect the relationship between telework satisfaction, subjective wellbeing, and self-reported performance.

#### Gender

Previous research has shown that telework leads to more work-family conflicts particularly for women (Karanikas & Cauchi, 2020), and that the intensity of working from home escalates dramatically with total hours worked (Dockery & Bawa, 2014). These findings are explained by gender role theory. The expectations of permeability of boundaries are much higher in women, reflecting societal expectations of women to be more present as parents and ‘housewives’ than men. Accordingly, working women might experience more difficulties with telework during lockdown because family and work were equally demanding.

#### Age

Recent studies offer mixed arguments and evidence about the role that age plays in telework. Thus, we included this control variable for exploratory testing (e.g., Nakrošienė et al., 2019).

#### Children

Participants reported if they had children under 18 years old. Having children is an important variable in telework studies (Kazekami, 2020). The presence of others could distract teleworkers to the point of decreasing their satisfaction and productivity (Baker et al., 2007). In the context of lockdown, it is even more important to control for this variable, as children were at home increasing the demands for parents to help with homeschooling, sometimes sharing the parents’ personal laptops, and having to care for their needs and demands while working.

#### Telework Experience

Participants reported if they had worked from home prior to the pandemic. Employees with prior telework experience might have more technical resources and professional competences to work from home (Carillo et al., 2020), and this may affect telework outcomes. Suitability of the working place at home was found to be one of the most important telework factors impacting different telework outcomes (Nakrošienė et al., 2019).

### Data Analyses

For data analyses we applied SPSS Statistics v. 27.0 to generate descriptive statistics and we used PROCESS v. 3.4 (Hayes, 2013) to test our hypotheses. We specifically used PROCESS Model 4 to compute the regression analyses with lagged effects, the confidence intervals (CIs) of the indirect effect of telework satisfaction on performance through wellbeing, and bootstrap tests with 5000 subsamples (Shrout & Bolger, 2002). We used PROCESS because this method is suitable for time-lagged data and it facilitates the estimation of CIs for indirect effects (Richey et al., 2016). Following Hayes (2013) recommendations, we tested the relationship between telework satisfaction and wellbeing (*path a*), wellbeing and performance (*path b*), the total effect of telework satisfaction on performance (*path c*), the direct effect of telework satisfaction on performance controlling for wellbeing (*path c’*), and the indirect effect of telework satisfaction on performance through wellbeing. We also controlled for age, gender, prior telework experience, and having children under 18 years old as covariates. Additionally, we used t-tests for complementary analyses.

## Results

Descriptive statistics including means, standard deviations and correlations are shown in Table 2. It should be noted that the mean WHO-five wellbeing score for our sample was 53.8 (SD = 18.6), almost 12 percent points lower during the pandemic compared to the norm in 2012 (Topp et al., 2015). Moreover and as expected, telework satisfaction was significantly and positively related with subjective wellbeing (r = 0.20; p = 0.030) and self-reported performance (r = 0.27, p = 0.004).

**Table 2:** Means, standard deviations and correlations (N = 111)

	M	SD	1	2	3	4	5	6
1. Telework satisfaction	4.52	.90	-					
2. Wellbeing	53.8	18.6	.20^*^	-				
3. Performance	3.80	.82	.27^**^	.27^**^	-			
4. Age	42.45	8.34	.11	.15	.09	-		
5. Gender	-	-	-.05	-.06	.06	.08	-	
6. Prior telework experience	-	-	-.02	.11	-.04	.03	-.45^**^	-
7. Children	-	-	-.34^**^	.11	.02	-.16	-.05	.12

Gender (1 = men; 2 = women), Prior telework experience (1 = no; 2 = yes), Children (1 = children < 18 years old; 0 = children > 18 years old or no children)

^*^ p < .05; ^**^p < .01

### Hypotheses Testing

**Fig. 1:**
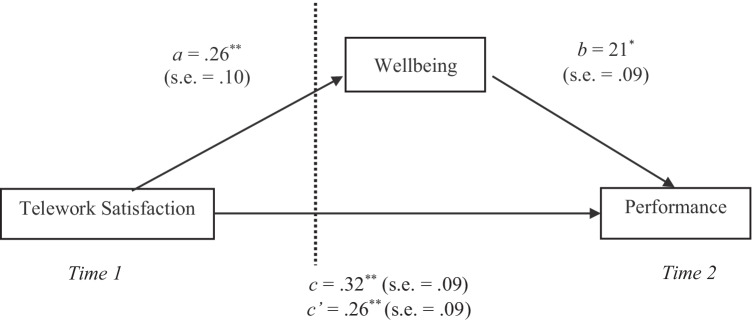
Direct and mediated relationships between telework satisfaction, wellbeing and performance

The results showed that telework satisfaction was positively associated with subjective wellbeing (*path a*: *β* = 0.26, p = 0.008) supporting H1; and subjective wellbeing was positively associated with performance (*path b*: *β* = 0.21, p = 0.029) supporting H3. The total effect of telework satisfaction on performance was significant (*path c*: *β* = 0.32, p = 0.001) and, after entering subjective wellbeing to the model, the beta weight associated with telework satisfaction decreased but remained significant (*path c’*: *β* = 0.26, p = 0.009), thus, supporting H2. In addition, the indirect effect of telework satisfaction on performance via subjective wellbeing was also significant (*β* = 0.06, boot SE = 0.03, boot 95% CI [0.0015, 0.1404]), confirming H4. To summarize, results from the study showed that there was an indirect relationship between high telework satisfaction and high performance. This association was partially mediated by higher levels of subjective wellbeing (Fig. 1). Additionally, we found that one control variable, ‘having children under 18 years old’, had significant effects on subjective wellbeing (β = 0.21, p = 0.031). Overall, the regression model explained 14.6% of the variance in performance (F (6, 104) = 2.96, p = 0.010). The magnitude of the effects is small-medium according to Cohen's (1992) cut-off points (r = 0.10, small; r = 0.30, medium; and r = 0.50, large), or moderate according to Acock’s (2014) criteria (β < 0.2, weak; 0.2 < β < 0.5, moderate; and β > 0.5 strong effect).

### Exploratory Analyses

In general, employees were satisfied with telework (M = 4.52, SD = 0.90). However, several results point at the relevance of the control variable ‘having children under 18 years old’. Besides the negative and significant correlation between this control variable and telework satisfaction, the regression model shows a negative and significant correlation between telework satisfaction and the variable ‘children under 18 years old’ (r = -0.34; p < 0.001). Additionally, the regression model shows that ‘having children under 18 years old’ had significant effects on subjective wellbeing (b = 0.41, s.e. = 0.19, p = 0.031).

We performed t-tests to explore the differences in telework satisfaction and subjective wellbeing between the group of employees with vs. without children under 18. On the one hand, telework satisfaction was significantly lower (t = 4.36, p < 0.001) for employees with children under 18 years (M = 4.19, SD = 0.92) than their counterparts (M = 4.88, SD = 0.79). On the other hand, subjective wellbeing was significantly higher (t = -2.14, p = 0.033) for employees with children under 18 (M = 3.65, SD = 0.91) than for employees without children under 18 (M = 3.41, SD = 1.10). In summary, telework satisfaction was lower when employees had children under 18, whereas their levels of subjective wellbeing were higher.

## Discussion

The goal of the present study was to explore the role of telework satisfaction in challenging times such as the COVID-19 lockdown when wellbeing was threatened in several ways, and to explore the effects of telework satisfaction on wellbeing and performance. By focusing on satisfaction with telework conditions, and not simply comparing satisfaction levels among teleworkers vs. non-teleworkers (e.g., Morilla-Luchena, et al., 2021), we go a step further questioning that telework is per se a source of job satisfaction. We highlight the importance of analyzing satisfaction with telework conditions suggesting that the telework experience is more individual than common across employees (Ipsen et al., 2021). Overall, we add to the literature of telework by providing empirical evidence that a key point for positive telework outcomes lies in the employee’s satisfaction with the virtual working conditions, and this may be the case during a crisis as well as under regular circumstances as we discuss next.

The first hypothesis proposed that telework satisfaction would increase the employees’ subjective wellbeing. Our results showed that employees satisfied with telework and its conditions presented higher levels of subjective wellbeing over time (H1 supported). These results are in line with the spillover hypothesis (Bakker & Demerouti, 2013) and the protective role played by work in the face of a crisis (Heir et al., 2021). Many areas in life were affected by lockdown (i.e. fears of infection, financial insecurity, job insecurity, sudden reduction in social life) thereby threatening previous levels of wellbeing. Our results show that satisfaction with telework had a positive influence on the employee’s emotional wellbeing during lockdown. Our sample had little previous experience with teleworking and most likely the sudden change came along with some stress to adapt to the new system. However, it seems that being able to (tele)work has given employees the chance to stay focused and busy with work, and the opportunity to keep in contact with colleagues and supervisors and benefitting from their support (Ortiz-Bonnín et al., 2016). Overall, our results show that satisfactory telework protected the employees’ subjective wellbeing.

Second, our results support the hypothesized positive effects of telework satisfaction on self-reported performance over time (H2 supported). This is consistent with the assumptions of the attitude-behavior link, whereby a positive attitude towards telework increases positive work behaviors, which in turn increase performance (Ostroff, 1992). It is also consistent with the assumptions of social exchange theory (Blau, 1964), which proposes that the employee will reciprocate satisfactory teleworking conditions with positive behaviors towards the organization.

Third, our findings showed that subjective wellbeing is positively associated with self-reported performance (H3 supported) and is in line with the happy-productive worker thesis (Zelenski et al., 2008). According to this result, general (non-related to work) wellbeing has a direct effect on performance. In a crisis context, it is important that organizations are aware of potential negative effects of ill-being or stress on performance. Heir et al.'s (2021) study recommended organizations to develop contingency plans for crisis management whereby managers are trained to understand expected stress reactions and their likely effects on a reduced capacity to work, and organizations can provide support to help employees stay well (mental and emotional wellbeing) and adapt faster to the new situation to be productive again.

Fourth, we found that the relationship between telework satisfaction and self-reported performance was partially mediated by subjective wellbeing (H4 supported). Therefore, telework satisfaction has a direct positive effect on self-reported performance (H2), but it also increases performance through its positive effect on general wellbeing (H4). If organizations want to maximize employee performance, they should create satisfactory teleworking conditions and support the employees’ subjective wellbeing, especially in times of crisis.

Finally, it is worth mentioning the significant results associated with ‘having children under 18 years old’ as a control variable. Having children under 18 implied less satisfaction with telework and higher levels of subjective wellbeing than employees without children under 18. Research prior to COVID19 already highlights that parents experience more daily joy but also more daily stress than nonparents (Deaton & Stone, 2014). However, working with children at home requires to simultaneously manage work and family demands, and reduces the chance to concentrate at work without interruptions (i.e. one of the main advantages claimed by telework advocates). The other side of the coin is that having children under 18 was positively related with subjective wellbeing. Thus, having children might alleviate parents’ feelings of social isolation due to lockdown.These findings are in line with previous research findings that having children at home while teleworking is likely to be both a ‘resource’ (compensating the loss of social interactions at work) and a ‘demand’ (increases home demands and workload; Shockley et al., 2021).

In summary, our results showed that satisfactory telework during COVID-19 lockdown increased wellbeing and performance, two key outcomes for organizational effectiveness. Furthermore, results supported the mediational role of wellbeing in the telework satisfaction- performance relationship. Although wellbeing scores seem to have decreased during the pandemic-induced lockdown compared to previously available data, the results obtained on the relationships between satisfactory telework, wellbeing and performance seem comparable to those in non—crisis situations.

This study contributed to the existing telework research stream in several ways. First, we add to the telework literature by providing empirical evidence highlighting that satisfactory telework is beneficial for both the employee’s wellbeing and performance and, subsequently, for the organization. Second, our prospective design to study the effects of satisfactory telework on subjective wellbeing and performance on telework represents a step-forward from previous cross-sectional research with only one time point of data collection. Third, we send a clear message to practitioners: If organizations want their teleworkers to be productive, they have to provide satisfactory working conditions and also take care of their employees’ wellbeing, which has been a widely neglected area of inquiry within the field of human resource management (Baptiste, 2008).

### Practical Implications

Our results underline the importance of telework satisfaction for subjective wellbeing and self-reported performance. This is relevant for two main reasons. First, teleworking is not only likely to continue after the pandemic, but it is expected to be predominant in the Post COVID-19 era (Grzegorczyk et al., 2021). Second, because it is critical for organizations to understand how telework satisfaction affects wellbeing and performance, two relevant outcomes for company success. What can management do to contribute to positive work and life outcomes? Offering telework as an alternative work arrangement may not be enough. Companies have to carefully design and evaluate telework conditions: provide adequate technical equipment for telework, ensure employees have physical working resources at home (e.g., Nakrošienė et al., 2019), and offer telework trainings, recommendations and policies about how to organize telework. Furthermore, organizations should overcome the disadvantages of telework uncovered during lockdown (e.g., loss of social contact with colleagues and supervisors and isolation due to fulltime telework) by measures such as implementing hybrid work models in the immediate future. Mixing days at the office with days at home could mitigate possible negative long-term effects of fulltime telework (Grzegorczyk et al., 2021).

To sum up, organizations can improve the success of telework by carefully designing specific working conditions for telework. The effort will not be in vain for two reasons: designing adequate telework conditions will prepare organizations for future crisis, and telework is here to stay, thus, it is more than a response to exceptional situations such as a lockdown. Finally, we encourage organizations to monitor the implementation of telework and to evaluate employee satisfaction with teleworking. In the same way, human resources departments should assess the personal and professional outcomes of teleworking through surveys or interviews and implement measures that help telework lead to wellbeing and performance.

### Limitations and Future Research

The present study has a few limitations to be considered when interpreting results and for future research. First, the generalization of the results is limited, because data for this study were collected through snowball sampling. Second, the sample was highly feminized (80%), and research with gender-balanced and larger samples is needed to study whether different groups (men and women) or functions (managers and employees) present similar or different effects of satisfaction with telework. Third, we used self-reports to assess the research variables and employees may be biased when rating, for example, their own performance. However, performance ratings by other people can also be problematic, because observers may lack adequate knowledge, because target behaviors may depend on unobservable mental processes, or because peers’ or managers’ ratings of performance may be subject to *halo effect* or their general impression of the employee (Viswesvaran et al., 2005; Warr & Nielsen, 2018). In our case, no other option than self-ratings was viable due to collection difficulties during the extraordinary crisis context and lockdown measures. Fourth, our data were gathered during lockdown and allowed studying short-term effects of satisfactory telework. Collecting longitudinal data after lockdown would have complemented our findings and allowed identifying potential positive long-term effects of telework adjustment (Carillo, 2020) and negative long-term effects of fulltime telework (i.e. feelings of social/professional isolation).

Finally, it was not possible to compare employees telework satisfaction, wellbeing, and performance with data collected before the lockdown. The results we obtained when studying ‘crisis-induced telework’ seem consistent with the findings in non-crisis times or ‘conventional telework’. Nonetheless, it is recommended that future studies compare results in a crisis context with those in conventional telework conditions.

## Conclusion

The health crisis (during the first COVID-19 lockdown) pushed employees in many jobs and industries into the digital era from one moment to the next. The conclusion of our study is that satisfactory telework predicted higher levels of subjective wellbeing and self-reported performance over time. This finding obtained in the context of ‘crisis-induced telework’ is consistent with previous research on the influence of satisfaction on wellbeing and performance. Telework satisfaction is important for wellbeing and performance during non-COVID-conditions as well. Wellbeing seems to be important for performance even (or especially) in times of crisis as it is in times of non-crisis. Organizations interested in employee wellbeing and performance should provide satisfactory telework conditions to allow employees to stay well, if they are to stay productive in the long run. This seems particularly important in crisis times and lockdown situations.
